# Egg yolk antibodies (IgY) against Bovine Leukemia Virus

**DOI:** 10.1186/1742-4690-11-S1-P46

**Published:** 2014-01-07

**Authors:** Cecilia Martínez, Gerónimo Gutiérrez, Irene Alvarez, Natalia Porta, Marina Lomónaco, Andrés Wigdorovitz, Pablo Chacana, Karina Trono

**Affiliations:** 1 Instituto de Virologia, INTA, Castelar, Argentina

## 

Bovine Leukemia Virus (BLV) is distributed worldwide and causes important economic losses on dairy farms. Currently, there are no effective vaccines or antivirals against BLV. Egg yolk antibodies (IgY) has many advantages over mammalian IgG. Despite the higher yields, they are non-invasively extracted from egg yolk, do not cross react against mammalian antigens or activate the mammalian complement system. In this work we evaluate the reactivity of Igy antibodies against Bovine Leukemia Virus p24 core protein and against the whole virus particle. Hens were immunized by intramuscular inoculation with purified p24 or the virus particle until the development of high antibody-titers. Total IgY was purified from egg yolks by ammonium sulfate precipitation. The purified egg yolk antibodies strongly reacted with BLV particles from a persistently infected cell line, with supernatants from ex vivo cultures of PBMCs from natural infected animals and also with purified p24 by both ELISA and Western blot. These data suggest that chicken IgY may be a suitable platform to produce large amounts of anti-BLV antibodies for diagnostic systems. Furthermore, the use of IgY for passive immunization against BLV infection should also be explored in order to develop new strategies to control the disease in cattle.

